# Target-enhanced double-pulse LIBS coupled with feature-fused CNN for mechanistic and interpretable coffee origin authentication

**DOI:** 10.1016/j.fochx.2026.103734

**Published:** 2026-03-10

**Authors:** Xiaoyong He, Kaiqiang Que, Tingrui Liang, Zhenman Gao, Zenghui Wang, Yufeng Li

**Affiliations:** a School of Telecommunications Engineering & Intelligentization, Dongguan University of Technology, Dongguan 523808, China; b Guangxi Key Laboratory of Information Functional Materials and Intelligent Information Processing, School of Physics & Electronics, Nanning Normal University, Nanning 530004, China; c School of Physics and Optoelectronics, South China University of Technology, Guangzhou 510640, China

**Keywords:** Coffee origin authentication, Double-pulse LIBS, Feature-fused CNN, SHAP, 1D Grad-CAM++, Trace element analysis

## Abstract

Coffee geographical origin authentication is critical for mitigating economically motivated adulteration, yet rapid trace-element analysis in complex organic matrices remains a significant challenge. This study establishes a novel synergistic framework integrating Potassium-assisted orthogonal Double-Pulse Laser-Induced Breakdown Spectroscopy (DP-LIBS) with a Feature-Fused CNN for precise coffee traceability. A high-purity KHCO₃ solid target was employed to facilitate plasma cross-coupling and secondary energy injection, significantly enhancing signal sensitivity. Surmounting the inherent bottlenecks of manual feature engineering, a Feature-Fused CNN architecture was constructed by concatenating normalized spectral data with statistical descriptors, enabling the autonomous extraction of hierarchical spatial-spectral patterns. The proposed model achieved a superior classification accuracy and F1-score of 99.00%, significantly outperforming traditional algorithms including XGBoost (95.75%), PLS-DA (92.50%), RF (86.50%), and KNN (75.75%). Robustness evaluation demonstrated that the CNN maintained high precision (>94%) even under severe noise interference (30 dB SNR). Furthermore, a dual-interpretability strategy was implemented to elucidate the decision logic: SHAP analysis was utilized to quantify feature contributions for traditional machine learning models, identifying key markers such as Fe, Cr, and Na; meanwhile, 1D Grad-CAM++ was applied to the Feature-Fused CNN to visualize wavelength-specific activation weights. The results reveal that the CNN's superior performance stems from recognizing the synergistic covariance of trace elements (Fe, Cr, Cu, and K) rather than isolated spectral peaks, providing a robust and mechanically interpretable strategy for food provenance verification.

## Highlights

• Potassium-assisted orthogonal DP-LIBS coupled with Feature-Fused CNN achieves 99.00% coffee origin classification accuracy, outperforming traditional algorithms.
• KHCO₃ target-enhanced orthogonal DP-LIBS realizes plasma cross-coupling, markedly boosting trace element signal sensitivity in complex coffee matrices.
• The Feature-Fused CNN fuses spectral and statistical features, retaining >94% accuracy under 30 dB SNR with exceptional anti-interference performance.
• Dual interpretability via SHAP and 1D Grad-CAM++ reveals model’s reliance on Fe/Cr/Cu/K synergistic covariance, addressing deep learning “black box” issues.
• This work establishes a generalizable paradigm for trace element analysis in complex food matrices, with broad industrial application potential.

## 1 Introduction

Coffee represents a pivotal commodity in global agricultural trade, with its commercial value and organoleptic quality being intrinsically linked to geographical origin(Tieghi et al., 2024). Specifically, *Coffea arabica* varieties are typically cultivated in high-altitude regions such as the Ethiopian Plateau, the Colombian Andes, and the Yunnan highlands, where environmental conditions favor the accumulation of chlorogenic and citric acids, resulting in characteristic floral and fruity flavor profiles. In contrast, *Coffea canephora* (Robusta) predominates in low-altitude areas like the Vietnamese Central Plains and Indonesian Java, characterized by higher concentrations of caffeine and trigonelline that contribute to a distinct bitterness (Mendes, Oliveira, Rodarte, Anjos, & Bell, 2024). However, this terroir-driven pricing mechanism is increasingly undermined by economically motivated adulteration, such as the mislabeling of Vietnamese Robusta as Colombian Arabica, which significantly erodes market trust (Mihailova et al., 2022). Although various analytical techniques have been employed to address this challenge, they face inherent limitations: sensory evaluation is prone to subjectivity (Soares, de Alencar, Chiarello, & de Oliveira, 2025); chromatographic and mass spectrometric methods (e.g., GC–MS and LC-MS), despite their precision, require complex and time-consuming sample preparation (Núñez et al., 2025); elemental and isotopic analyses (e.g., ICP-MS) often lack sufficient granularity for fine-scale discrimination (Valentin & Watling, 2013); spectroscopic techniques such as NIR and THz exhibit poor model generalization (Fu, Ren, & Sun, 2024; Mutz et al., 2023); and NMR, while non-destructive, struggles with scalability and high instrumentation costs (Alvarenga et al., 2025).

Laser-Induced Breakdown Spectroscopy (LIBS) has established itself as a premier analytical technique due to its rapid, multi-element detection capabilities and minimal sample preparation requirements (Silva et al., 2017; Silva, Milori, Neto, Ferreira, & Ferreira, 2019). However, conventional single-pulse LIBS (SP-LIBS) often suffers from limited sensitivity, poor limits of detection (LOD), and signal instability caused by matrix effects and plasma shielding (Gautier et al., 2004, Gautier et al., 2005). To address these challenges, Double-Pulse LIBS (DP-LIBS) has been widely adopted as an effective plasma modulation method, significantly enhancing emission intensity through mechanisms such as increased ablated mass, elevated plasma temperature, and elongated plasma lifetime (Noll, Sattmann, Sturm, & Winkelmann, 2004). For instance, Ahmed and Baig achieved a ∼300-fold intensity increase in aluminum alloys using a collinear arrangement (Ahmed & Baig, 2009), while Stratis et al. reported a 30-fold signal enhancement and a 5000 K temperature rise in orthogonal pre-ablation configurations. Furthermore, Wang et al. demonstrated that orthogonal reheating DP-LIBS offers superior stability, reducing the Relative Standard Deviation (RSD) to 2% compared to 5% in SP-LIBS (Wang et al., n.d.). In agricultural and food applications, Nicolodelli et al. utilized DP-LIBS to achieve a 155-fold enhancement for magnesium in fertilizers by increasing the ablated mass (Nicolodelli et al., 2016), and Peng et al. successfully combined reheating DP-LIBS with machine learning for high-accuracy chromium detection in rice leaves (Peng et al., 2019). Despite these advancements, trace element analysis in complex organic matrices like coffee remains challenging due to strong background interference and low ionization efficiency. To overcome this, this study introduces a novel Potassium-assisted orthogonal DP-LIBS system employing a high-purity KHCO₃ solid target. By synchronizing vertical sample ablation with horizontal target excitation to facilitate plasma cross-coupling and secondary energy injection, this method significantly suppresses background noise and enhances trace element sensitivity to the ppb level for precise coffee origin tracing.

Regarding data processing, traditional machine learning methods like KNN and RF are widely used but often struggle with high-dimensional spectral data due to the curse of dimensionality and the loss of local features(Biau, 2012; Boateng, Otoo, & Abaye, 2020). In recent years, the synergistic integration of LIBS with advanced deep learning, multimodal fusion, and explainable artificial intelligence (XAI) has emerged as a frontier research direction in food traceability and environmental monitoring. Recent studies demonstrate the superiority of deep architectures in handling complex matrices. For instance, Iroshan et al. developed the DLIBS-FFNet deep learning framework, which fuses dimensionality-reduced features to achieve superior robustness over traditional methods in the anatomical classification and heavy metal quantification of legumes (Iroshan, Aizezi, & Liu, 2025). Simultaneously, to transcend the limitations of single-source spectral information, Meng et al. proposed a multimodal fusion strategy (LIBS-FLIPA) combining LIBS with laser-induced plasma acoustic signals. By leveraging frame segmentation algorithms to extract complementary features, they significantly enhanced classification accuracy in challenging environments (Meng, Gao, Ye, & Liu, 2025), highlighting the potential of heterogeneous feature fusion. Furthermore, the increasing complexity of models has driven the demand for interpreting “black box” algorithms. Ye et al. successfully constructed a DTEWD-XGBoost-SHAP framework for single-particle aerosol analysis, utilizing SHAP values to quantitatively elucidate the regulatory mechanisms of key chemical components on physical properties (Ye et al., 2025). These advancements underscore the necessity of simultaneously achieving deep feature extraction and model interpretability in high-performance classification tasks.

Consequently, this study establishes a synergistic framework that integrates feature engineering with deep fusion architecture to transcend the performance bottlenecks of conventional methodologies. Specifically, a Feature-Fused CNN model is constructed by concatenating min-max normalized spectral data with four statistical descriptors—peak count, mean peak intensity, mean signal intensity, and standard deviation—into a unified, information-rich input vector. This design enables the autonomous extraction of hierarchical spatial-spectral patterns via convolutional layers, thereby significantly enhancing robustness in coffee authentication. The primary objectives of this research are threefold: (1) developing a KHCO₃-enhanced orthogonal DP-LIBS system with synchronized sample-target motion for augmenting trace-element sensitivity and mitigating background interference; (2) implementing the spectral-statistical feature fusion framework via the Feature-Fused CNN, which is rigorously benchmarked against traditional machine learning models including KNN, RF, PLS-DA, and XGBoost; and (3) elucidating critical spectral markers for geographical authentication through SHAP analysis and Grad-CAM++ visualization, bridging the gap between elemental signatures and soil-climatic factors to provide mechanistic insights into the classification logic.

## 2 Materials and methods

### 2.1 Sample preparation and data acquisition

**Fig. 1:**
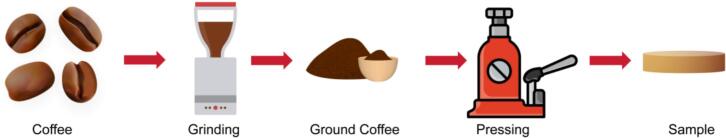
Schematic diagram of coffee sample preparation process.

Medium-roasted coffee beans representing four distinct geographical origins—Yunnan, Colombia, Kenya, and Ethiopia—were utilized in this study. Specifically, beans originating from Yunnan and Ethiopia were sourced from Lujiazui Coffee (Baoshan) Co., Ltd.; Colombian beans were purchased from Linyi Chenka Trading Co., Ltd.; and Kenyan beans were obtained from Lipuyao International Trade (Beijing) Co., Ltd. The samples were pulverized using a high-speed multifunctional grinder operating at 35,000 rpm for 2 min. The resulting powder was sieved to pass through a 200-mesh screen and subsequently homogenized. Aliquots of the coffee powder (3.0 g) were compressed into pellets (30 mm diameter; 3.5 mm thickness) under 20 tons of hydraulic pressure. For calibration purposes, KHCO₃ target discs (6.0 g; 30 mm diameter; 4.0 mm thickness) were prepared from analytical-grade powder under identical pressure conditions to ensure surface planarity for optimal laser coupling (Fig. 1). To mitigate hygroscopic effects and ensure chemical stability, all prepared pellets and targets were stored in a desiccator maintained at <10% relative humidity prior to analysis.

**Fig. 2:**
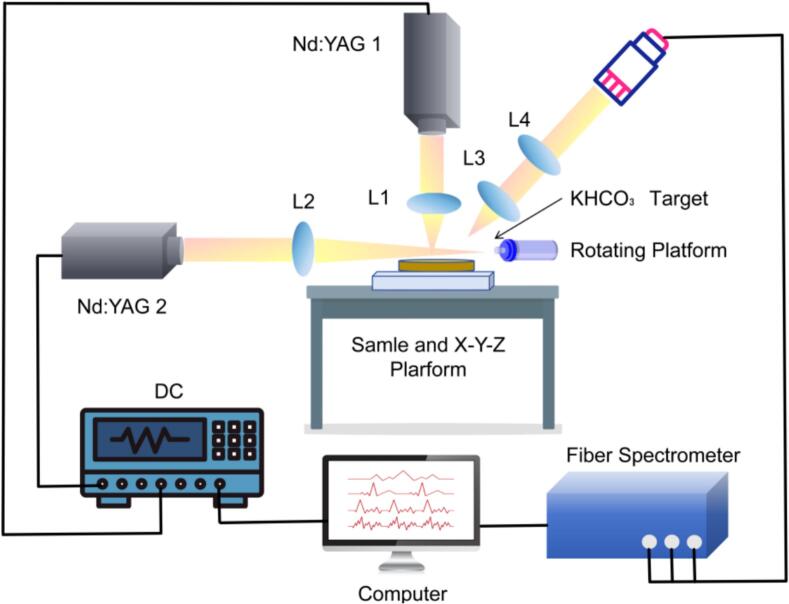
Schematic of the DP-LIBS system.

Spectral data acquisition was performed using a target-enhanced orthogonal DP-LIBS system. The first laser pulse (20 mJ) vertically ablated the coffee pellet to generate primary plasma and preheat the sample surface. After a 6 μs delay, the second pulse (60 mJ) horizontally irradiated the KHCO₃ target, exciting potassium-enriched plasma that coupled with the coffee-derived plasma to prolong lifetime and enhance electron-impact excitation efficiency. To ensure homogeneous ablation distribution, the target underwent linear translation at 0.5 mm/s while the coffee pellet rotated at 5 rpm (Fig. 2). Spectrometers were configured with a gate delay of 1.5 μs and gate width of 2 ms. Each spectrum represented an average of five consecutive laser pulses. For each geographical origin, four independent pellets were analyzed, with 50 high-resolution spectra acquired per pellet, yielding a total dataset of 800 independent spectral files. Raw spectral data spanned 200–550 nm, each spectrum comprising 6145 wavelength-intensity data points. All experiments were conducted under controlled environmental conditions (25 ± 1 °C, 50 ± 5% relative humidity). The heavy-metal concentrations presented in Table 1 were validated via ICP-MS to ensure data reliability.

**Table 1:** Coffee Elemental concentrations (μg/kg).

Sample Name	Kenya	Ethiopia	Colombia	Yunnan
Na	13,421.43	8167.56	8481.90	11,527.01
K	11,465,852.26	17,195,739.59	12,627,503.01	15,561,426.92
Ca	344,222.74	532,753.06	357,557.97	505,804.29
Cr	372.91	93.54	209.15	118.12
Mn	38,195.74	51,395.28	14,992.81	78,978.92
Fe	23,921.60	15,891.07	12,189.72	22,542.08
Cu	10,888.66	1539.17	15,010.54	9088.13
As	2.52	<0.1	<0.1	<0.1
Cd	1.78	36.40	0.54	19.04
Hg	<0.1	<0.1	<0.1	<0.1

### 2.2 Spectral preprocessing methods

Addressing the dimensional heterogeneity between spectral wavelength coordinates and intensity magnitudes is a prerequisite for ensuring numerical stability and accelerating convergence in gradient-based optimization. To strictly prevent data leakage and ensure the model's generalization capability on unseen data, a rigorous data isolation protocol was implemented prior to any global normalization. Specifically, the dataset was partitioned into training and independent test sets, with all preprocessing parameters derived exclusively from the training data.

Consequently, a global standardization framework was established across the training spectral domain $D_{\mathrm{train}}={\left\{\left(\lambda_{i},I_{i}\right)\right\}}_{i=1}^{N_{\mathrm{train}}}$. To map the disparate ranges of spectral position and radiometric response into a unified latent space, global boundaries $V_{\min}$ and $V_{\max}$ were defined based solely on the extrema of the training dataset:

(1) $$V_{\min}=\min\left(\min\left(\lambda \in D_{\mathrm{train}}\right),\min\left(I\in D_{\mathrm{train}}\right)\right)$$

(2) $$V_{\max}=\max\left(\max\left(\lambda \in D_{\mathrm{train}}\right),\max\left(I\in D_{\mathrm{train}}\right)\right)$$

The normalized spectral vector $x^{\prime }$ for both training and testing samples is obtained via a Min-Max scaling transformation using these pre-computed training parameters:

(3) $$x{'}_{i}=\frac{x_{i}-V_{\min}}{V_{\max}-V_{\min}+\epsilon }$$

where ϵ is a regularization constant to prevent singularity. Crucially, for the validation and test sets, the same $V_{\min}$ and $V_{\max}$ values derived from the training set were applied, ensuring that no information from the test data influenced the feature scaling process.

Following normalization, a fused spectral signature vector $S_{\mathrm{fused}}$ was generated by the element-wise summation of the normalized wavelength and intensity vectors ($S_{\mathrm{fused}}=\lambda^{\prime }+I^{\prime }$). Complementing this continuous spectral representation, discrete statistical descriptors were extracted to capture salient morphological landmarks and global distributional characteristics. A peak detection algorithm was employed to isolate significant diagnostic features, defined as local maxima exceeding a dynamic amplitude threshold of $0.8\times \max\left(I^{\prime }\right)$. The set of indices corresponding to these peaks, denoted as P, allows for the derivation of the peak count ($N_{p}$) and the mean peak intensity ($\mu_{p}$):

(4) $$N_{p}=|P|$$

(5) $$\mu_{p}=\left\{\begin{array}{ll} \frac{1}{N_{p}}\sum_{k\in P}I{'}_{k} & \text{if}\;N_{p}>0\\ 0 & \text{if}\;N_{p}=0 \end{array}\right)$$

Furthermore, to characterize the overall signal energy and variability—which often correlate with sample concentration and scattering properties—the global mean intensity ($\mu_{\mathrm{global}}$) and global standard deviation ($\sigma_{\mathrm{global}}$) were computed over all N spectral channels:

(6) $$\mu_{\text{global}}=\frac{1}{N}\sum_{i=1}^{N}I{'}_{i}$$

(7) $$\sigma_{\text{global}}=\sqrt{\frac{1}{N}\sum_{i=1}^{N}{\left(I{'}_{i}-\mu_{\text{global}}\right)}^{2}}$$

The final high-dimensional feature vector F is constructed by concatenating the fused spectral signature with these statistical moments:

(8) $$F=S_{\text{fused}}⊕{\left[N_{p},\mu_{p},\mu_{\text{global}},\sigma_{\text{global}}\right]}^{⊤}$$

This comprehensive vector design serves a multi-faceted purpose: it preserves the full spectral detail essential for chemical fingerprinting while simultaneously capturing morphological nuances through peak-based parameters. Moreover, by quantifying the distributional patterns of the signal via statistical metrics, the proposed feature set enhances the model's robustness against spectral variations. Ultimately, this approach provides the classifier with a standardized, high-information-density input, significantly elevating classification performance.

### 2.3 Classification methods

#### 2.3.1 Conventional machine learning algorithms

RF is an ensemble classification method that constructs multiple decision trees and aggregates their predictions through majority voting or averaged probabilities (Fawagreh, Gaber, & Elyan, 2014). Its core mechanism employs bootstrap sampling to generate diverse training subsets while randomly selecting feature subsets at each node split, reducing inter-tree correlation and enhancing generalization. This approach makes RF robust for classification and regression tasks involving high-dimensional data and noisy environments (Ye, Wu, Huang, Ng, & Li, 2013).

KNN is a non-parametric classification algorithm that classifies unlabeled samples based on common similarity metrics, including various distance measures such as Euclidean distance, Manhattan distance, and generalized Minkowski distance (Zhang, Li, Zong, Zhu, & Cheng, 2017). It identifies the k closest training samples and assigns the majority class through voting. The optimal k value balances overfitting and underfitting, typically determined through cross-validation. Despite its simplicity, KNN exhibits sensitivity to high-dimensional data, necessitating feature selection or dimensionality reduction techniques to enhance efficiency in spectral analysis (Wang, An, Chen, Li, & Alterovitz, 2015).

PLS-DA integrates dimensionality reduction with classification in a supervised approach. It extracts latent variables to maximize inter-class variance, projects high-dimensional features into a low-dimensional space, and constructs a linear discriminant model(Lee, Liong, & Jemain, 2018). Particularly effective for scenarios with limited samples but high feature dimensionality such as spectral data, PLS-DA avoids multicollinearity issues by optimizing principal components while balancing feature interpretability and classification accuracy(Saccenti & Timmerman, 2016).

XGBoost iteratively trains gradient-boosted decision trees by optimizing weighted loss functions and fitting residuals. Innovations include L1/L2 regularization for model complexity control, parallel computing support, and sparse data handling, significantly improving training speed and generalization(Chen, 2016). Its robustness in class-imbalanced and nonlinear problems makes it ideal for complex spectral classification(Caramês et al., 2025).

Bayesian optimization is an intelligent hyperparameter tuning algorithm leveraging probabilistic modeling to enhance model performance. It constructs Gaussian process-based probability distributions for objective functions such as validation metrics, iteratively guiding search direction by dynamically refining surrogate models through prior evaluations. The algorithm balances exploration of unknown regions and exploitation of known optima using acquisition functions like Expected Improvement(Snoek, Larochelle, & Adams, 2012). In contrast to traditional grid search, Bayesian optimization leveraging the Tree-structured Parzen Estimator (TPE) algorithm demonstrates significant computational efficiency by navigating high-dimensional parameter spaces to identify optimal configurations with fewer evaluations. In this study, the hyperparameters of the RF, KNN, PLS-DA, and XGBoost models were rigorously optimized over 50 iterations. A composite weighted metric was utilized as the objective function, integrating the F1-score (weight: 0.5) to address precision-recall trade-offs in class-imbalanced scenarios and accuracy (weight: 0.5) for a holistic assessment of predictive capability. The detailed hyperparameter search ranges and configurations are presented in Table 2.

**Table 2:** Optimized hyperparameter tuning ranges for classification models.

Model	Hyperparameter	Tuning range	Options
RF	n_estimators	50–500	Integer
	max_depth	5–30	Integer
	min_samples_split	2–20	Integer
	min_samples_leaf	1–10	Integer
KNN	n_neighbors	1–30	Integer
	weights	–	[‘uniform’, ‘distance’]
	p	1–5	Integer
PLS-DA	n_components	2–20	Integer
XGBoost	n_estimators	50–500	Integer
	max_depth	3–10	Integer
	learning_rate	0.01–0.3	Logarithmic Sampling
	subsample	0.6–1.0	Float
	colsample_bytree	0.6–1.0	Float
	min_child_weight	1–10	Integer
	gamma	0–5	Float

#### 2.3.2 Convolutional neural network with statistical feature fusion

CNN emulate the hierarchical processing of biological visual systems to establish multilevel feature abstraction capabilities. The core architecture comprises convolutional layers extracting spatial features through local receptive fields with weight-sharing mechanisms, nonlinear ReLU activation functions enhancing model expressiveness, pooling layers preserving salient features via downsampling while improving translational invariance, and fully connected layers integrating high-level abstract features for classification decisions. This hierarchical stacking enables CNN to autonomously learn low-to-high-order feature representations from raw data, proving particularly effective for processing spatially correlated spectral signals(Li, Liu, Yang, Peng, & Zhou, 2021).

**Fig. 3:**
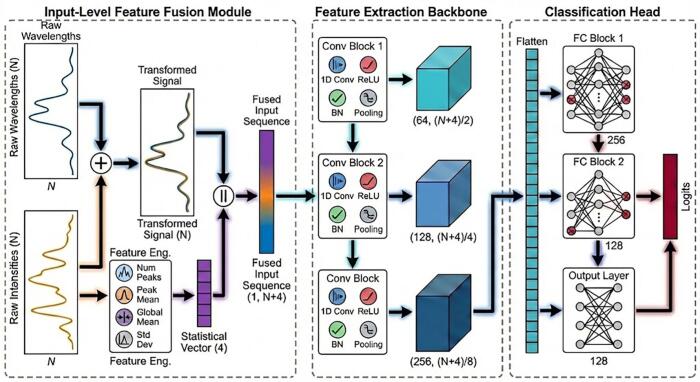
Schematic diagram of the feature-fused CNN architecture.

A feature-fused CNN architecture was developed for spectral data analysis, as illustrated in Fig. 3. The model input consists of a composite feature vector constructed by concatenating the normalized spectral profile with four engineered statistical descriptors: peak count, mean peak intensity, mean signal intensity, and standard deviation. The network backbone comprises three sequential convolutional blocks with increasing channel depths of 64, 128, and 256, respectively. Each block performs a 1D convolution (kernel size = 3, padding = 1), followed by ReLU activation, batch normalization, and max pooling (kernel size = 2) to extract hierarchical spectral features. The resulting feature maps are flattened and propagated through a multi-stage fully connected classifier consisting of dense layers with 256, 128, and 4 nodes. To mitigate overfitting, dropout regularization is applied after the first two dense layers at rates of 0.3 and 0.2, respectively. The model was trained for 50 epochs using the Adam optimizer (learning rate = 1 × 10^−4^) with a Cross-Entropy loss function, modulated by a cosine annealing learning rate scheduler and a batch size of 32.

### 2.4 Experimental design and computational framework

Ensuring the rigor and unbiased nature of the experimental evaluation, the raw dataset was initially stratified into an independent test set and a development set. Approximately 10% of the samples were strictly sequestered as the independent test set, which was completely excluded from model training and hyperparameter optimization to serve exclusively for the final verification of generalization performance. The remaining 90% constituted the development set, utilized to execute a 5-fold cross-validation scheme. In this process, data were randomized and partitioned into five mutually disjoint subsets, with four subsets selected for training and one for validation during each iteration to comprehensively assess model stability and generalization. Accuracy and Macro F1-score were established as the core evaluation metrics, while confusion matrices were utilized to visually elucidate the detailed distribution characteristics of classification outcomes.

All computational workflows were executed on a high-performance workstation running Windows 11 Professional, equipped with an Intel Core i5-12500T processor, 64 GB DDR5 RAM, and an NVIDIA RTX A2000 GPU. The software environment operated within Python 3.12.9 hosted by PyCharm Professional 2023.2.8. Traditional machine learning models utilized scikit-learn 1.6.1, whereas the CNN architecture leveraged PyTorch 2.6.0 with CUDA 12.4 for GPU acceleration. Furthermore, systematic hyperparameter optimization was managed via Optuna 4.3.0, with matplotlib 3.10.0 and seaborn 0.13.2 employed for high-resolution data visualization.

## 3 Results and discussion

### 3.1 Coffee spectral profiles

**Fig. 4:**
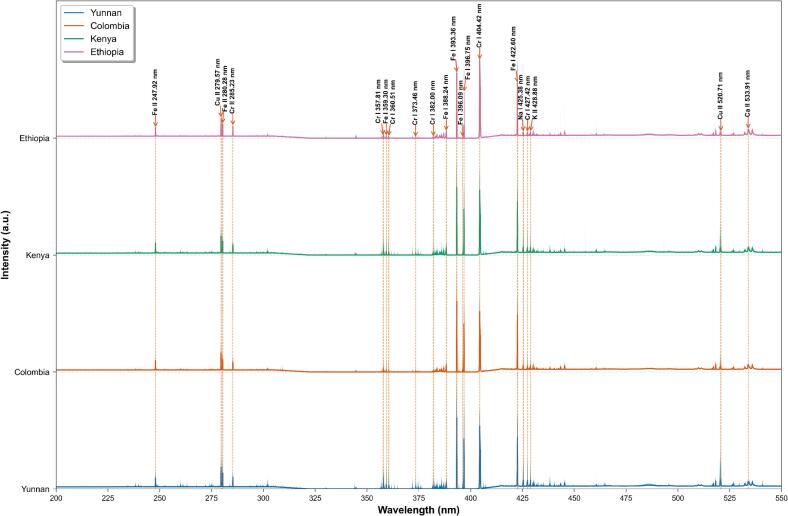
Spectral profiles of coffee varieties.

LIBS was employed to analyze the elemental composition of coffee samples from multiple geographical origins. As illustrated in Fig. 4, the average LIBS spectra across 200–550 nm exhibited structural similarities, indicating shared fundamental elemental constituents. Key emission lines were identified by referencing the U.S. National Institute of Standards and Technology atomic spectra database (NIST ASD, https://physics.nist.gov/PhysRefData/ASD/lines_form.html) (Cai et al., 2023), Dominant peaks revealed elemental signatures such as Fe, Cr, Na. Despite these overarching similarities, discernible variations in emission intensities at specific wavelengths were observed among origin-specific samples. The inherent spectral complexity characterized by densely overlapping emission lines from multiple elements precluded reliable visual differentiation of geographical origins. Consequently, while spectral fingerprints establish a foundation for origin discrimination, their interpretation necessitates advanced chemometric approaches. Subsequent machine learning or deep learning analysis is essential for extracting latent origin-related information to achieve robust classification.

### 3.2 Analysis of dimensionality reduction visualizations

Scrutinizing the intrinsic manifold structure of high-dimensional raw LIBS spectral data necessitated the implementation of three complementary dimensionality reduction techniques including PCA, t-SNE, and UMAP. Preceding the dimensionality reduction, raw spectral intensities underwent *Z*-score normalization to mitigate amplitude scaling effects and ensure equal contribution of all spectral variables. It is imperative to note that these dimensionality reduction algorithms were employed exclusively for exploratory data visualization and qualitative cluster assessment; they were not utilized as input features for the subsequent supervised classification models. PCA was selected as the linear benchmark to extract orthogonal directions of maximum variance for global topology preservation while quantifying the variance contribution of each component(Maćkiewicz & Ratajczak, 1993; Unnikrishnan et al., 2013). Addressing the complex non-linear relationships inherent in spectral signatures, t-SNE was utilized to maintain local neighborhood structures by minimizing the Kullback-Leibler divergence between probability distributions in high- and low-dimensional spaces(Linderman, Rachh, Hoskins, Steinerberger, & Kluger, 2019; Lopes, Neto, & Martins, 2020). Additionally, UMAP was deployed based on topological data analysis theory to balance the preservation of global structure and local neighborhoods while offering superior computational efficiency(McInnes, Healy, & Melville, 2018; Sainburg, McInnes, & Gentner, 2021). Reproducibility and optimal visualization were guaranteed through precise hyperparameter configuration where PCA extracted the first two principal components, t-SNE was initialized with a perplexity of 30 and 1000 iterations, and UMAP was configured with 15 neighbors and a minimum distance of 0.1. Deterministic execution for all stochastic algorithms was enforced by fixing the random state to 42.

**Fig. 5:**
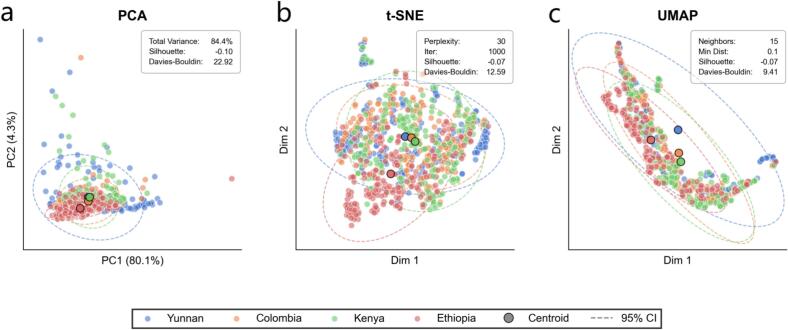
Visualization of dimensionality-reduced coffee spectra: (a) PCA; (b) t-SNE; (c) UMAP.

As visualized in Fig. 5a, the linear projection via PCA indicates that the global variance is predominantly captured by the first principal component. PC1 accounts for 80.1% of the total variance while PC2 explains 4.3% which cumulatively represents 84.4% of the spectral information. Despite this high explained variance, the score plot demonstrates severe overlapping among the four coffee origins. The centroids of Yunnan, Colombia, Kenya, and Ethiopia samples are tightly clustered within the central region and exhibit extensive intersection of their 95% confidence ellipses without distinct decision boundaries. Quantitative assessment corroborates this visual indistinguishability as the Silhouette Coefficient is −0.10 and the Davies-Bouldin Index is 22.92. The negative Silhouette score is particularly diagnostic as it reveals that the average intra-class distance exceeds the inter-class distance in the linear space. This confirms that the dominant direction of variance in the raw spectra is driven by factors orthogonal to the chemical differences associated with coffee varieties.

In pursuit of elucidating latent local structures, t-SNE (Fig. 5b) and UMAP (Fig. 5c) were employed to probe non-linear manifolds. Both methods unveiled a striking phenomenon where the data spontaneously organized into approximately four distinct agglomerations. However, visual inspection of the spatial distribution reveals that these clusters are highly heterogeneous. Within each isolated island, the samples from the four different geographical classes are intermixed rather than stratified by coffee type, and the centroids of the respective classes remain spatially proximal within the dense central regions. Although the structural compactness improved significantly as evidenced by the reduction of DBI from 22.92 in PCA to 12.59 in t-SNE and 9.41 in UMAP, the Silhouette Coefficients remained negative at −0.07 for both methods. This metric discrepancy highlights a critical data characteristic where non-linear algorithms successfully detected strong local manifolds as indicated by the lower DBI, but the basis of this clustering does not correspond to the target labels as indicated by the negative Silhouette score. The observation that t-SNE and UMAP detect distinct data separations unrelated to coffee types points to the prevalence of systematic background variance. This stratification is attributed to physical spectral responses arising from variations in sample granularity, packing density, or minor environmental fluctuations such as humidity and temperature during acquisition. These physical baseline shifts generate a variance magnitude that overshadows the subtle chemical fingerprints of the cultivars. Consequently, unsupervised algorithms which prioritize maximizing global variance like PCA or preserving topological proximity based on raw Euclidean distances like t-SNE and UMAP inherently cluster data based on these dominant physical attributes. This finding provides the fundamental rationale for the supervised Feature-Fused CNN framework proposed in this study. Unlike unsupervised methods, the convolutional layers of the CNN function as adaptive semantic filters, effectively suppressing this high-variance physical background noise to extract the nuanced, discriminative geochemical features required for accurate coffee origin classification.

### 3.3 Comparative performance and interpretative attribution of conventional machine learning

#### 3.3.1 Benchmarking classification efficacy and error distribution patterns

**Fig. 6:**
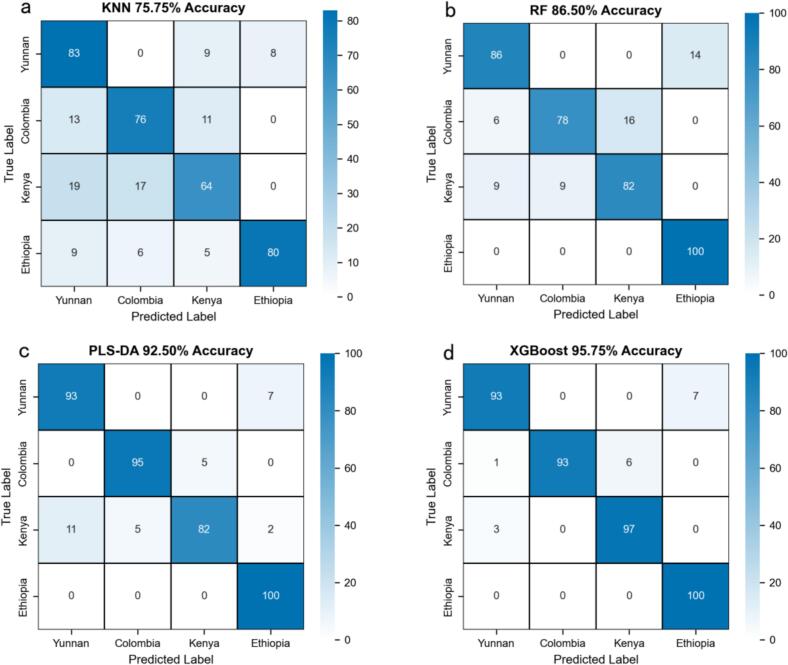
Confusion matrices of conventional machine learning algorithms: (a) KNN; (b) RF; (c) PLS-DA; (d) XGBoost.

Comparative analysis of the four machine learning algorithms for coffee variety classification revealed significant performance disparities, as quantified in Fig. 6. Aggregated five-fold cross-validation results generated confusion matrices, with final metrics representing mean values across validation rounds. KNN (Fig. 6a) exhibited the lowest accuracy at 75.75%, demonstrating substantial inter-varietal confusion including 17% of Yunnan samples misclassified as Colombia or Kenya, 24% of Colombia samples erroneously assigned to Yunnan or Kenya, 36% of Kenya samples misclassified as Yunnan or Colombia, and 20% of Ethiopian samples misclassified. RF (Fig. 6b) achieved 86.50% accuracy with flawless identification of Ethiopian samples but displayed a 14% misclassification rate for Yunnan primarily confused with Ethiopia, a 22% misclassification rate for Colombia primarily confused with Kenya, and an 18% misclassification rate for Kenya assigned to Yunnan or Colombia. PLS-DA (Fig. 6c) attained 92.50% accuracy, perfectly classifying Ethiopian samples but misassigning 7% of Yunnan as Ethiopia, 5% of Colombia as Kenya, and 18% of Kenya across the other three origins. XGBoost (Fig. 6d) achieved optimal performance among traditional models at 95.75% accuracy, showing exceptional discriminative power including 100% accuracy for Ethiopia, 93% for Yunnan with 7% misclassified as Ethiopia, 93% for Colombia, and 97% for Kenya with 3% misclassified as Yunnan. The superior precision of ensemble-based XGBoost and dimensionality-reduced PLS-DA stems from their enhanced capacity to model complex spectral relationships and capture subtle varietal differences. Analysis of the prevalent misclassification patterns suggests links to geographical origins, with confusion between the high-altitude origins Yunnan and Ethiopia observed across multiple models potentially reflecting overlapping elemental profiles, and confusion between the mid-altitude origins Colombia and Kenya, possibly arising from comparable spectral characteristics influenced by environmental factors. These performance gaps are not merely a byproduct of algorithmic design but are deeply tied to two interrelated core factors: first, the intrinsic spectral variability of coffee varieties, driven by geo-environmental factors such as soil composition, climate, and altitude, shapes the elemental and chemical composition of coffee beans, giving rise to unique spectral fingerprints that serve as the fundamental basis for varietal classification; second, algorithms differ significantly in their ability to decode these fingerprints—XGBoost, an ensemble model, effectively models nonlinear spectral patterns by aggregating weak learners, PLS-DA retains discriminative features through dimensionality reduction, and RF balances complexity with interpretability, while KNN, a distance-based method, struggles to disentangle subtle environment-induced spectral variations, resulting in inferior performance. The combined effect of these factors explains the performance gradient from KNN to XGBoost, and the higher precision of ensemble-based XGBoost and dimensionality-reduced PLS-DA ultimately stems from their enhanced capacity to model complex spectral relationships and capture subtle varietal differences.

#### 3.3.2 SHAP-based spectral feature attribution and geochemical linkages

Aiming to enhance the interpretability and transparency of machine learning classification models, SHAP analysis was performed on the optimal test sets of four trained models: RF, XGBoost, KNN, and PLS-DA. The top-10 significant spectral feature wavelengths extracted from the data served as input variables, and SHAP analysis quantified the contribution of individual spectral features to classification decisions across coffee varieties. TreeExplainer was used for tree-based algorithms RF and XGBoost, as it leverages the hierarchical splitting logic of decision trees to compute exact SHAP values efficiently, avoiding the approximations needed for non-tree models and ensuring alignment with the sequential feature interactions inherent to these ensemble methods(Deb & Smith, 2021), while KernelExplainer was applied to distance-based and linear models KNN and PLS-DA. Since these models lack tree structures, KernelExplainer uses a kernel method to approximate SHAP values, making it compatible with KNN's distance-driven similarity calculations and PLS-DA's linear latent variable projections, thus maintaining methodological consistency with their underlying mechanisms(Bifarin & Fernández, 2024).

**Fig. 7:**
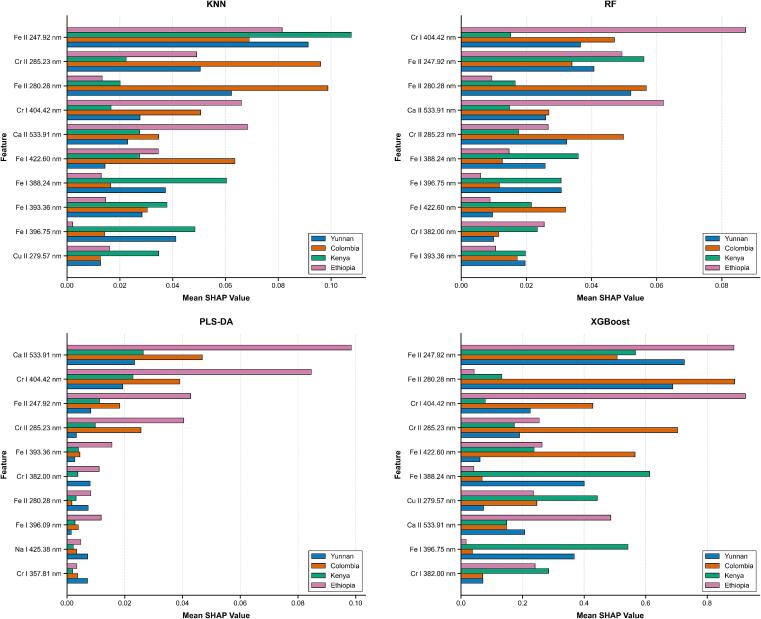
TOP features SHAP values: (a) KNN; (b) RF; (c) PLS-DA; (d) XGBoost.

As visualized in Fig. 6, the SHAP value analysis illuminated the internal inference mechanisms of the four traditional machine learning models, revealing that while algorithmic strategies differ, geographical discrimination consistently relies on specific trace element fingerprints (Fe, Cr, Na, Cu). KNN (Fig. 7a), which is sensitive to local manifold structures, prioritized Cr II (397.96 nm) and Na II (292.35 nm) as primary discriminators; it effectively characterized Yunnan coffee and Colombia coffee through shared chromium features, while isolating Kenya coffee via a unique sodium signature and differentiating Ethiopia coffee using a dominant chromium profile. The ensemble-based RF (Fig. 7b) captured global spectral variances, establishing Fe I (300.75 nm) as a ubiquitous predictor for Yunnan coffee and Colombia coffee, with critical secondary differentiation provided by Fe II (315.28 nm) for Kenya coffee and Cr II (397.96 nm) for Ethiopia coffee. PLS-DA (Fig. 7c), defining linear boundaries, similarly identified Cr II (397.96 nm) as the dominant separator for Ethiopia coffee and Yunnan coffee, while employing Fe I (405.32 nm) and Cr I (403.33 nm) to characterize Colombia coffee and Kenya coffee. The gradient-boosted XGBoost (Fig. 7d) exhibited a distinct high-gain feature selection strategy, assigning exceptional importance to Fe I (300.75 nm) for classifying Colombia coffee and Yunnan coffee; uniquely, it diverged by identifying Fe II (315.28 nm) as a critical marker for Kenya coffee, while characterizing Ethiopia coffee through a combined profile of Fe I (300.75 nm) and Cu II (300.47 nm). However, despite identifying these discrete markers, traditional machine learning models are inherently limited by their reliance on shallow, linear, or distance-based feature interactions. They often lack the capacity to autonomously extract hierarchical spatial-spectral dependencies or effectively suppress background noise interference in complex matrices, which restricts their robustness compared to deep learning architectures that learn holistic spectral representations.

### 3.4 Feature-fused CNN: algorithmic superiority and mechanistic insights

#### 3.4.1 Evaluation of classification robustness and computational resource efficiency

**Fig. 8:**
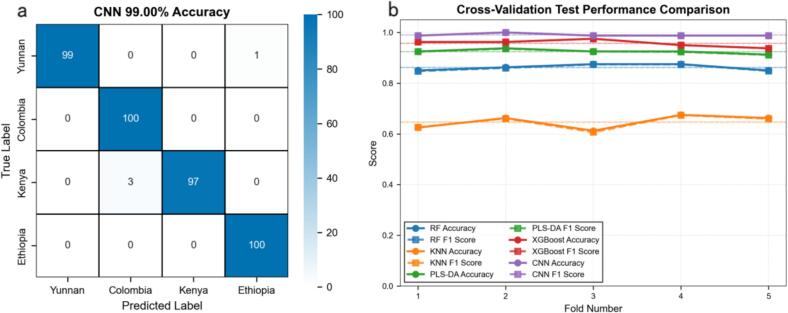
CNN performance metrics: (a) Confusion matrix; (b) Comparative line plot of model accuracy.

Performance evaluation of the proposed CNN algorithm demonstrated exceptional efficacy in classifying coffee varieties, achieving an average test set accuracy of 99.00% and an F1-score of 99.00%, which significantly outperformed all conventional machine learning benchmarks. The CNN model surpassed the best-performing traditional model, XGBoost (95.75%), as well as PLS-DA (92.50%), RF (86.50%), and KNN (75.75%), establishing a robust superiority in discriminating complex spectral signatures. Confusion matrix analysis (Fig. 8a) further corroborated this precision, revealing 99% accuracy for Yunnan samples, 97% for Kenyan samples, and a flawless 100% classification rate for both Colombian and Ethiopian origins. The stability of the model was confirmed via five-fold cross-validation (Fig. 8b), where the CNN consistently maintained near-perfect accuracy across all folds, contrasting sharply with the variability observed in distance-based methods like KNN. This performance leap stems from the CNN's end-to-end architecture, which unifies spatial-spectral feature learning to autonomously capture subtle, non-linear inter-varietal variations that manual feature engineering fails to resolve.

While the CNN architecture achieved the highest classification accuracy, a rigorous quantitative evaluation of computational resources reveals a distinct trade-off between predictive precision and algorithmic complexity. As presented in Table 3, the deep learning model incurred a substantial computational cost during the training phase, requiring an average of 1194.11 s, which is significantly higher than the rapid training times of shallow algorithms such as PLS-DA (0.92 s) and XGBoost (30.65 s). In terms of deployment efficiency, linear and tree-based models demonstrated superior inference speeds; specifically, PLS-DA and XGBoost achieved ultra-low latencies of 0.0715 ms and 0.1867 ms per sample, respectively, benefiting from their computationally efficient matrix projections and decision tree traversals. In contrast, the CNN exhibited a higher average inference latency of 4.9406 ms due to the dense matrix operations inherent in multi-layer convolutions. However, this latency remains negligible for real-time industrial screening applications, and notably, the CNN maintained a highly compact memory footprint (0.0995 MB), significantly outperforming the memory-intensive PLS-DA (56.4547 MB). Consequently, the increased computational expenditure during the training phase is a justifiable investment. It supports an end-to-end learning paradigm that autonomously extracts hierarchical spatial-spectral features, thereby transcending the performance ceilings imposed by the manual feature engineering and linear assumptions of traditional models to achieve robust, high-precision authentication.

**Table 3:** Comparison of performance metrics among different models.

Model	Test set accuracy (%)	Test set F1-score (%)	Average training time (s)	Average training memory (MB)	Average inference time per sample (ms)
RF	86.50 ± 2.09	86.32 ± 2.16	8.6927 ± 0.0857	14.0435 ± 0.0012	0.3127 ± 0.0056
KNN	75.75 ± 2.42	75.56 ± 2.51	0.0064 ± 0.0010	0.0652 ± 0.0000	34.8779 ± 0.7733
PLS-DA	92.50 ± 0.79	92.39 ± 0.77	0.9180 ± 0.0554	56.4547 ± 0.0001	0.0715 ± 0.0068
XGBoost	95.75 ± 1.27	95.74 ± 1.28	30.6517 ± 0.5189	0.0123 ± 0.0010	0.1867 ± 0.0176
CNN	99.00 ± 0.50	99.00 ± 0.50	1194.1127 ± 31.5318	0.0995 ± 0.0518	4.9406 ± 0.1424

#### 3.4.2 1D Grad-CAM++ visualization: Elucidating the spectral logic of geographical origin

The 1D Grad-CAM++ technique was implemented to decode the decision-making process of the Feature-Fused CNN and elucidate wavelength-specific contributions to geographical classification. Unlike conventional approaches relying on global average pooling, Grad-CAM++ utilizes pixel-wise weighting coefficients to resolve unweighted localization issues, thereby enhancing the identification of multiple occurrences of class-discriminative features such as repeating emission lines(Cai et al., 2023; Chattopadhay, Sarkar, Howlader, & Balasubramanian, 2018).

Mathematically, let $Y^{c}$ represent the classification score for a specific coffee origin c prior to the softmax layer, and $A^{k}$ denote the k-th feature map in the final convolutional layer. The importance weight $w_{k}^{c}$ for the k-th feature map is computed by integrating the gradients with a weighting coefficient $\alpha_{i}^{\mathrm{kc}}$, formulated as:

(9) $$w_{k}^{c}=\sum_{i}\alpha_{i}^{\mathrm{kc}}\cdot \text{ReLU}\left(\frac{\partial Y^{c}}{\partial A_{i}^{k}}\right)$$

Here, $\alpha_{i}^{\mathrm{kc}}$ represents the weighting coefficient for the i-th spectral channel in the k-th feature map. This coefficient is derived using a closed-form solution involving second- and third-order partial derivatives of the class score with respect to the feature maps, ensuring a more refined sensitivity to spectral intensity variations:

(10) $$\alpha_{i}^{\mathrm{kc}}=\frac{\frac{\partial^{2}Y^{c}}{{\left(\partial A_{i}^{k}\right)}^{2}}}{2\frac{\partial^{2}Y^{c}}{{\left(\partial A_{i}^{k}\right)}^{2}}+\sum_{a}A_{a}^{k}\frac{\partial^{3}Y^{c}}{{\left(\partial A_{a}^{k}\right)}^{3}}}$$

The final class-discriminative localization map $L_{\text{Grad}-\mathrm{CAM}++}^{c}$ is obtained by the linear combination of weighted feature maps, followed by a ReLU activation to suppress negative contributions:

(11) $$L_{\text{Grad}-\mathrm{CAM}++}^{c}=\text{ReLU}\left(\sum_{k}w_{k}^{c}A^{k}\right)$$

As visualized in Fig. 8, this methodology enables the precise localization of critical spectral regions. To rigorously quantify the reliability of these spectral fingerprints, the Standard Deviation of the generated activation weights was analyzed across samples. The Standard Deviation serves as a statistical metric for spectral consistency. A low value at a specific wavelength indicates that the CNN consistently activates this spectral region across varying samples of the same origin, identifying it as a robust geochemical marker immune to sample heterogeneity. Conversely, a high value suggests substantial intra-class variability in feature expression, potentially attributable to fluctuations in trace element concentrations, matrix effects, or environmental noise during data acquisition. This dual analysis combining gradient-based localization with statistical stability offers a methodological framework that connects deep learning predictions with spectrochemical interpretability.

**Fig. 9:**
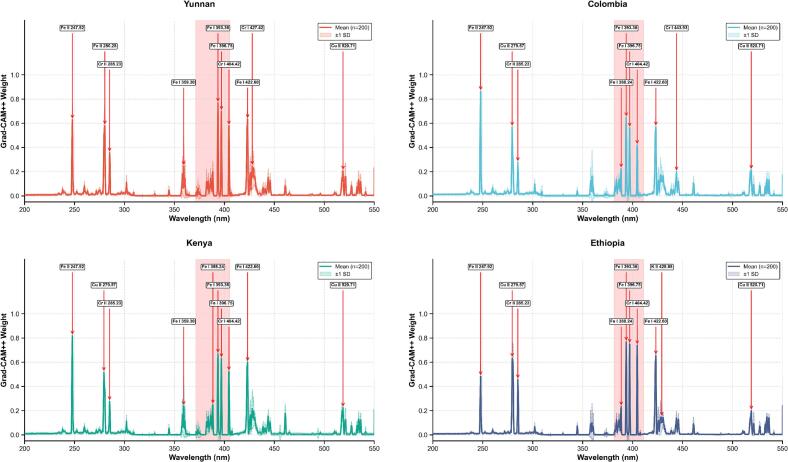
1D Grad-CAM++ visualizations for CNN models: (a) Yunnan; (b) Colombia; (c) Kenya; (d) Ethiopia. Each colored solid line shows the mean weight per wavelength for samples of a given origin. The shaded area of the same color shows the corresponding standard deviation.

The 1D Grad-CAM++ visualization analysis decoded the wavelength-specific contributions to the CNN model's decision-making process, elucidating the spectral logic underpinning geographical classification. For Yunnan coffee (Fig. 9a), the classification was driven by a complex profile where the model prioritized Fe I emissions (393.36, 396.75, 422.60, 359.30 nm) and Fe II lines (247.92, 280.28 nm), further reinforced by specific Cr I (404.42, 427.42 nm), Cr II (285.23 nm), and K II (428.88 nm) signatures as diagnostic markers. Colombian coffee (Fig. 9b) was characterized primarily by a prominent response at Fe II (247.92 nm), supported by a broad spectrum of Fe I activations (393.36, 422.60, 396.75, 388.24 nm); notably, the model identified unique Cu II (279.57, 520.71 nm) emissions combined with Cr I (404.42, 443.93 nm) and Cr II (285.23 nm) to distinguish this origin. Similarly, Kenyan coffee (Fig. 9c) displayed a strong reliance on the Fe II (247.92 nm) emission, yet it was differentiated by the combined influence of specific Fe I peaks (393.36, 396.75, 422.60, 388.24, 359.30 nm) and a distinct interplay of Cr I (404.42 nm), Cr II (285.23 nm), and Cu II (279.57 nm). In contrast, Ethiopian coffee (Fig. 9d) highlighted a unique multi-element fingerprint where Cr I (404.42 nm) emerged as a critical identifier alongside dominant Fe I emissions (393.36, 396.75, 422.60 nm); the concurrent prioritization of Cu II (279.57, 520.71 nm), Cr II (285.23 nm), and K II (428.88 nm), with a relatively lower emphasis on Fe II compared to other origins, suggests a distinct geochemical signature for this region. Quantitative analysis across all varieties indicates that the 391.2–408.8 nm spectral interval consistently accumulated the highest importance weights. These results demonstrate that while ubiquitous Fe I and Fe II emissions serve as general tracers for the organic matrix, the specific variations in Cr, Cu, and K emissions enable precise geographical discrimination correlated with local soil composition and climatic conditions, confirming that the CNN captures meaningful geochemical fingerprints rather than noise. Crucially, these findings demonstrate that the model authenticates geographical origin by recognizing the synergistic covariance of multiple elements rather than relying on isolated spectral peaks. This multi-parametric dependency ensures that the classification is governed by the holistic geochemical fingerprint involving Fe, Cr, Cu, and K. Consequently, this mechanism effectively filters out single-source contamination such as sporadic iron spikes caused by machinery wear because these artificial signals lack the requisite correlation with other terroir-specific markers, thereby guaranteeing the specificity and reliability of the authentication.

### 3.5 Robustness evaluation under noise interference

A systematic noise injection stress test was implemented to evaluate model reliability under simulated environmental interference. Gaussian white noise at varying signal-to-noise ratios ranging from the original clean signals down to 20 dB was superimposed onto the independent test set spectra. This procedure simulates progressively harsh measurement conditions often encountered in real-world deployment scenarios. The evaluation utilized the ensemble of models derived from the stratified five-fold cross-validation scheme, and the reported metrics represent the mean accuracy with standard deviations across the folds to ensure statistical rigor and eliminate data artifacts.

**Fig. 10:**
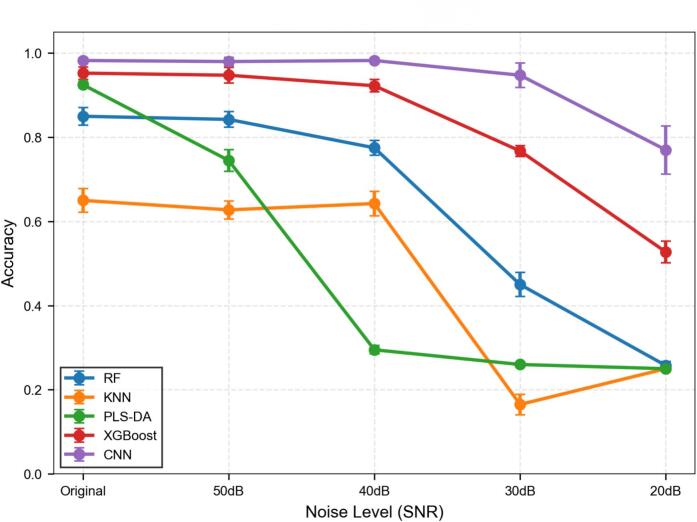
Robustness evaluation of classification models under varying noise levels.

As shown in Fig. 10, the robustness analysis reveals that the Feature-Fused CNN architecture exhibits exceptional resilience compared to traditional machine learning baselines. Conventional models experience a precipitous decline in classification accuracy when the signal-to-noise ratio falls below 40 dB, whereas the proposed deep learning model maintains a stable performance plateau with accuracy exceeding 94% even at a moderate noise level of 30 dB. This superior stability stems from the dual-stream architecture that effectively fuses spectral morphological features with statistical descriptors, where the convolutional layers function as adaptive filters to suppress high-frequency noise while preserving critical low-frequency characteristic peaks. Crucially, these results carry broader implications for the model's adaptability to complex food matrices beyond mere environmental noise resilience. In industrial processing, the introduction of additives such as sugar or milk typically induces spectral baseline fluctuations and non-linear matrix effects that are analogous to the signal degradation simulated by high-intensity noise. Therefore, the model's sustained high precision under low-SNR conditions serves as a robust proxy validation for its capability to decouple authentic geographical origin fingerprints from the spectral interferences caused by complex matrices, highlighting its potential applicability for processed coffee products.

## 4 Conclusions

This study successfully validates a novel synergistic framework integrating Potassium-assisted orthogonal DP-LIBS with a Feature-Fused CNN to address the critical challenge of rapid, non-destructive coffee geographical authentication. By employing a high-purity KHCO₃ solid target for plasma cross-coupling alongside a deep architecture that concatenates normalized spectral data with statistical descriptors, the proposed system effectively surmounted the sensitivity limitations of conventional atomic spectroscopy. The Feature-Fused CNN not only achieved a state-of-the-art classification accuracy of 99.00%, significantly outperforming traditional algorithms including XGBoost, PLS-DA, RF, and KNN, but also demonstrated exceptional robustness against signal degradation, maintaining high precision even under simulated matrix interferences. Crucially, this research addresses the intrinsic opacity of deep learning models by implementing a dual-interpretability strategy: SHAP analysis quantified feature contributions for traditional machine learning benchmarks, identifying key markers such as Fe and Cr, while 1D Grad-CAM++ provided unprecedented mechanistic insights into the CNN, visualizing wavelength-specific activation weights. These visualizations confirmed that the model's superior decision-making logic relies on recognizing the synergistic covariance of specific trace elemental fingerprints (Fe, Cr, Cu, and K) rather than isolated spectral peaks, thereby ensuring the geochemical validity of the classification. Future investigations should focus on applying this technology to the fine-scale provenance tracing of high-value agricultural products within a single nation, thereby rigorously evaluating the method's resolution limits under minimal geographical variance. Furthermore, advancing the miniaturization of this framework for portable LIBS instrumentation will foster a transition towards mechanistically transparent and reliable real-time food forensics.

## CRediT authorship contribution statement

**Xiaoyong He:** Writing – review & editing, Supervision, Resources, Project administration, Funding acquisition, Conceptualization. **Kaiqiang Que:** Writing – original draft, Software, Methodology, Investigation, Formal analysis. **Tingrui Liang:** Validation, Software, Formal analysis, Data curation. **Zhenman Gao:** Visualization, Software, Funding acquisition, Data curation. **Zenghui Wang:** Methodology, Investigation, Formal analysis, Data curation. **Yufeng Li:** Methodology, Formal analysis, Data curation.

## Declaration of competing interest

The authors declare that they have no known competing financial interests or personal relationships that could have appeared to influence the work reported in this paper.

## Data Availability

Data will be made available on request.

## References

[bb0005] Ahmed R., Baig M.A. (2009). A comparative study of single and double pulse laser induced breakdown spectroscopy. Journal of Applied Physics.

[bb0010] Alvarenga Y.A., Boness H.V.M., Sarmento C.S.A.G., Lemos O.L., Matsumoto S.N., Boffo E.F. (2025). Exploring Bahia’s coffee diversity: NMR spectroscopy and chemometrics unveil regional variations. Food Chemistry.

[bb0015] Biau G. (2012). Analysis of a random forests model. The Journal of Machine Learning Research.

[bb0020] Bifarin O.O., Fernández F.M. (2024). Automated machine learning and explainable AI (AutoML-XAI) for metabolomics: Improving cancer diagnostics. Journal of the American Society for Mass Spectrometry.

[bb0025] Boateng E.Y., Otoo J., Abaye D.A. (2020). Basic tenets of classification algorithms K-nearest-neighbor, support vector machine, random forest and neural network: A review. Journal of Data Analysis and Information Processing.

[bb0030] Cai Z., Huang Z., He M., Li C., Qi H., Peng J., Zhang C. (2023). Identification of geographical origins of Radix Paeoniae Alba using hyperspectral imaging with deep learning-based fusion approaches. Food Chemistry.

[bb0035] Caramês E.T., de Moraes-Neto V.F., Bertozzi B.G., da Silva L.P., Villa J.E., Pallone J.A., Correa B. (2025). Identification of *Fusarium sambucinum* species complex by surface-enhanced Raman spectroscopy and XGBoost algorithm. Food Chemistry.

[bb0040] Chattopadhay A., Sarkar A., Howlader P., Balasubramanian V.N. (2018). 2018 IEEE winter conference on applications of computer vision (WACV).

[bb0045] Chen T. (2016).

[bb0050] Deb D., Smith R.M. (2021). Application of random forest and SHAP tree explainer in exploring spatial (in) justice to aid urban planning. ISPRS International Journal of Geo-Information.

[bb0055] Fawagreh K., Gaber M.M., Elyan E. (2014). Random forests: From early developments to recent advancements. Systems Science & Control Engineering: An Open Access Journal.

[bb0060] Fu Y., Ren Y., Sun D.-W. (2024). Novel analysis of food processes by terahertz spectral imaging: A review of recent research findings. Trends in Food Science & Technology.

[bb0065] Gautier C., Fichet P., Menut D., Lacour J.-L., L'Hermite D., Dubessy J. (2004). Study of the double-pulse setup with an orthogonal beam geometry for laser-induced breakdown spectroscopy. Spectrochimica Acta Part B: Atomic Spectroscopy.

[bb0070] Gautier C., Fichet P., Menut D., Lacour J.-L., L'Hermite D., Dubessy J. (2005). Quantification of the intensity enhancements for the double-pulse laser-induced breakdown spectroscopy in the orthogonal beam geometry. Spectrochimica Acta Part B: Atomic Spectroscopy.

[bb0075] Iroshan A., Aizezi N., Liu Y. (2025). Rapid anatomical classification and lead contamination analysis in edible legumes using novel LIBS–deep learning frameworks. Journal of Food Composition and Analysis.

[bb0080] Lee L.C., Liong C.-Y., Jemain A.A. (2018). Partial least squares-discriminant analysis (PLS-DA) for classification of high-dimensional (HD) data: A review of contemporary practice strategies and knowledge gaps. Analyst.

[bb0085] Li Z., Liu F., Yang W., Peng S., Zhou J. (2021). A survey of convolutional neural networks: Analysis, applications, and prospects. IEEE Transactions on Neural Networks and Learning Systems.

[bb0090] Linderman G.C., Rachh M., Hoskins J.G., Steinerberger S., Kluger Y. (2019). Fast interpolation-based t-SNE for improved visualization of single-cell RNA-seq data. Nature Methods.

[bb0095] Lopes M.A.D.S., Neto A.D.D., Martins A.D.M. (2020). Parallel t-sne applied to data visualization in smart cities. IEEE Access.

[bb0100] Maćkiewicz A., Ratajczak W. (1993). Principal components analysis (PCA). Computers & Geosciences.

[bb0105] McInnes L., Healy J., Melville J. (2018).

[bb0110] Mendes G.d.A., Oliveira M.A.L.d., Rodarte M.P., Anjos V.d.C.d., Bell M.J.V. (2024). Determination of Arabica and Robusta species in blends of roasted coffee by Mid Infrared spectroscopy in association with mixture design. Food Chemistry Advances.

[bb0115] Meng H., Gao W., Ye Y., Liu Y. (2025). Multimodal LIBS-FLIPA fusion with frame segmentation for robust plastic classification via advanced LIPA processing. Optics Letters.

[bb0120] Mihailova A., Liebisch B., Islam M.D., Carstensen J.M., Cannavan A., Kelly S.D. (2022). The use of multispectral imaging for the discrimination of Arabica and Robusta coffee beans. Food Chemistry: X.

[bb0125] Mutz Y.S., do Rosario D., Galvan D., Schwan R.F., Bernardes P.C., Conte-Junior C.A. (2023). Feasibility of NIR spectroscopy coupled with chemometrics for classification of Brazilian specialty coffee. Food Control.

[bb0130] Nicolodelli G., Senesi G.S., de Oliveira Perazzoli I.L., Marangoni B.S., Benites V.D.M., Milori D.M.B.P. (2016). Double pulse laser induced breakdown spectroscopy: A potential tool for the analysis of contaminants and macro/micronutrients in organic mineral fertilizers. Science of the Total Environment.

[bb0135] Noll R., Sattmann R., Sturm V., Winkelmann S. (2004). Space- and time-resolved dynamics of plasmas generated by laser double pulses interacting with metallic samples. Journal of Analytical Atomic Spectrometry.

[bb0140] Núñez N., Moret E., Lucci P., Moret S., Saurina J., Núñez O. (2025). SPME-GC–MS and chemometrics for coffee characterization, classification and authentication. Microchemical Journal.

[bb0145] Peng J., He Y., Jiang J., Zhao Z., Zhou F., Liu F. (2019). High-accuracy and fast determination of chromium content in rice leaves based on collinear dual-pulse laser-induced breakdown spectroscopy and chemometric methods. Food Chemistry.

[bb0150] Saccenti E., Timmerman M.E. (2016). Approaches to sample size determination for multivariate data: Applications to PCA and PLS-DA of omics data. Journal of Proteome Research.

[bb0155] Sainburg T., McInnes L., Gentner T.Q. (2021). Parametric UMAP embeddings for representation and semisupervised learning. Neural Computation.

[bb0160] Silva T.V., Hubinger S.Z., Gomes Neto J.A., Milori D.M.B.P., Ferreira E.J., Ferreira E.C. (2017). Potential of Laser Induced Breakdown Spectroscopy for analyzing the quality of unroasted and ground coffee. Spectrochimica Acta Part B: Atomic Spectroscopy.

[bb0165] Silva T.V., Milori D.M.B.P., Neto J.A.G., Ferreira E.J., Ferreira E.C. (2019). Prediction of black, immature and sour defective beans in coffee blends by using Laser-Induced Breakdown Spectroscopy. Food Chemistry.

[bb0170] Snoek J., Larochelle H., Adams R.P. (2012). Practical Bayesian optimization of machine learning algorithms. Advances in Neural Information Processing Systems.

[bb0175] Soares C.A.L., de Alencar E.R., Chiarello M.D., de Oliveira L.d.L. (2025). Unraveling the impact of coffee fermentation: Interactions among processing variables and their effects on sensory quality. Trends in Food Science & Technology.

[bb0180] Tieghi H., Pereira L.d.A., Viana G.S., Katchborian-Neto A., Santana D.B., Mincato R.L., Bueno P.C.P. (2024). Effects of geographical origin and post-harvesting processing on the bioactive compounds and sensory quality of Brazilian specialty coffee beans. Food Research International.

[bb0185] Unnikrishnan V.K., Choudhari K.S., Kulkarni S.D., Nayak R., Kartha V.B., Santhosh C. (2013). Analytical predictive capabilities of Laser Induced Breakdown Spectroscopy (LIBS) with Principal Component Analysis (PCA) for plastic classification. RSC Advances.

[bb0190] Valentin J.L., Watling R.J. (2013). Provenance establishment of coffee using solution ICP-MS and ICP-AES. Food Chemistry.

[bb0195] Wang A., An N., Chen G., Li L., Alterovitz G. (2015). Accelerating wrapper-based feature selection with K-nearest-neighbor. Knowledge-Based Systems.

[bb0200] Wang, Q., Wang, J.-G., Liang, Y.-x., Chen, X.-l., Wu, B., Ni, Z.-b., & Dong, F.-z. Investigation on emission spectra of reheating and pre-ablation dual-pulse laser-induced breakdown spectroscopy. 8201, 625–633. doi:10.1117/12.907258.

[bb0205] Ye Y., Li J., Aizezi N., Wang Z., Li L., Deng L., Liu Y. (2025). Single-particle decoding of aerosol pollutants size-composition relationships: An interpretable XGBoost-SHAP framework with DTEWD-enhanced SPAMS analysis. Journal of Hazardous Materials.

[bb0210] Ye Y., Wu Q., Huang J.Z., Ng M.K., Li X. (2013). Stratified sampling for feature subspace selection in random forests for high dimensional data. Pattern Recognition.

[bb0215] Zhang S., Li X., Zong M., Zhu X., Cheng D. (2017). Learning k for knn classification. ACM Transactions on Intelligent Systems and Technology (TIST).

